# *Rhodiola crenulata* extract counteracts the effect of hypobaric hypoxia in rat heart *via* redirection of the nitric oxide and arginase 1 pathway

**DOI:** 10.1186/s12906-016-1524-z

**Published:** 2017-01-07

**Authors:** Shih-Wei Hsu, Tsu-Chung Chang, Yu-Kuan Wu, Kuen-Tze Lin, Li-Shian Shi, Shih-Yu Lee

**Affiliations:** 1 Department of Neurosurgery, Taichung Armed Forces General Hospital, Taichung, Taiwan; 2 Department of Biochemistry, National Defense Medical Center, Taipei, Taiwan; 3 Department of Radiation Oncology, Tri-Service General Hospital, National Defense Medical Center, Taipei, Taiwan; 4 Department of Biotechnology, National Formosa University, Yunlin, Taiwan; 5 Graduate Institute of Aerospace and Undersea Medicine, National Defense Medical Center, P.O. Box 90048-514, Nei-Hu 114, Taipei, Taiwan, R.O.C.

**Keywords:** *Rhodiola crenulata*, Hypobaric hypoxia, Heart, Nitric oxide, Arginase 1

## Abstract

**Background:**

*Rhodiola crenulata* is traditionally used as a folk medicine in Tibet for preventing high-altitude illnesses, including sudden cardiac death (SCD). The cardio-protective effects of *Rhodiola crenulata* root extract (RCE) against hypoxia *in vivo* have been recently confirmed. However, the way in which RCE produces these effects remains unclear. The present study is designed to confirm the protective effects of RCE on the heart in acute hypobaric hypoxia exposure and examine the mechanisms by which this occurs.

**Methods:**

Sprague–Dawley (SD) rats were pretreated with or without RCE and then exposed to a simulated altitude of 8000 m in a hypobaric hypoxia chamber for 9 h. The expression of cardiac arginase 1 (Arg-1) and endothelial nitric oxide synthase (eNOS) and the activity of associated signaling pathways was examined.

**Results:**

Hypoxia reduced cardiac eNOS phosphorylation and increased Arg-1 expression, but both responses were reversed by RCE pre-treatment. In addition, RCE decreased the hypoxia-induced oxidative stress markers of reactive oxygen species (ROS) production, malondialdehyde (MDA) level, and protein carbonyl content. Furthermore, RCE protected cardiomyocytes from hypoxia-induced cardiac apoptosis and restored the phosphorylation level of AKT and p38 MAPK as well as the superoxide dismutase 2 (SOD2) content in hypoxic animals.

**Conclusion:**

The findings provide evidence that the effects of *Rhodiola crenulata* against altitude illness are partially mediated by modulation of eNOS and Arg-1 pathways in the heart.

**Electronic supplementary material:**

The online version of this article (doi:10.1186/s12906-016-1524-z) contains supplementary material, which is available to authorized users.

## Background

Acute exposure to high altitude involves a hypobaric hypoxic environment. This may initiate pulmonary arterial vasoconstriction, increased vascular resistance, pulmonary hypertension, and high-altitude pulmonary edema (HAPE) in individuals. These events may even have lethal consequences [1]. In addition to the pulmonary alterations, sudden cardiac death (SCD) is another common cause of death that is associated with vasoconstriction and pulmonary hypertension and even more rapid lethality during high-altitude activities [2]. Hypobaric hypoxia is considered as a cardiac stressor and may lead to some cardiovascular disorders such as myocardial infarction (MI) and pulmonary hypertension-induced right ventricular (RV) dysfunction [3]. In individuals with heart disease, hypoxic conditions also decrease the ischemic threshold and increase the arrhythmia frequency [2]. Thus, the impact of hypobaric hypoxia on the heart has been a focus of investigation in recent years.

Nitric oxide (NO), a crucial regulator of vascular tone and blood pressure, is regarded as a cardiac protector [4]. Many hypoxia-induced disorders, including endothelial dysfunction, pulmonary artery vasoconstriction, and pulmonary hypertension (PH) are highly associated with impairment of the endothelial nitric-oxide synthase (eNOS) pathway and NO production [5, 6], especially in individuals who are prone to high-altitude illness [7].

Arginase, an important enzyme in the urea cycle, has been reported to be involved in many cardiovascular disorders such as PH, atherosclerosis, MI, and congestive heart failure [8]. Once activated, arginase competes with eNOS and reacts with L-arginine, resulting in reduced NO availability. Thus, arginase plays a crucial role in adaptation to hypoxia [9]. Reactive oxygen species (ROS) are also associated with pulmonary artery vasoconstriction and offsetting of NO signaling [10]. The hypoxia-induced ROS burst in the heart causes oxidative damage to biomolecules (DNA, proteins, and lipids), which may depress myocardial contraction and dysfunction [11].

*Rhodiola crenulata*, a popular folk medicine for anti-altitude sickness in Tibet [12], has been recently reported as having cardio-protective effects on hypoxia *in vivo* [13, 14] and in rodent models of acute exhaustive injury [15]. However, the detailed mechanisms by which RCE protects against hypoxia remain unclear. We have previously shown that RCE alleviates hypoxia-induced pulmonary edema in rats [16]. This effect was mediated via a decrease in the level of endothelin-1 (ET-1), a natural counterpart of NO in vascular function. In the present study, we have investigated whether RCE exerts cardio-protective effects against hypobaric hypoxic conditions via redirection of the NO and arginase 1 (Arg-1) pathway.

## Methods

### Preparation and Quality Control of RCE

Preparation and analysis of RCE were described in a previous study by our group [17]. Briefly, *Rhodiola crenulata* (Hook. f. & Thomson) H. Ohba roots were obtained from Chuang Song Zong Pharmaceutical Co., Ltd (Kaohsiung, Taiwan). A voucher specimen (NDMCP no.1000901) was deposited in the National Defense Medical Center. The identity of *R. crenulata* was confirmed using the Plant List database. 2.0 kg dry powder was extracted with 95% ethanol and then yielded 320.24 g RCE after condensation under reduced pressure. The drug-extract ratio was 6.25:1. To identify the content of bioactive marker salidroside in RCE, HPLC was conducted. The 10.0 mg of RCE dissolved in 1 ml of methanol was analyzed by HPLC with a Lichro CART^R^ RP-18e (4.0 mm × 250 mm i.d., 5 μm; Merck, Germany) column. The samples were eluted with a H_2_O-acetonitrile gradient system for 60 min at a flow rate of 1.0 ml/min. The analytes were quantified with a wavelength of 223 nm. The calibration curve was linear within a range of 0.125–1.0 mg/ml (R^2^ = 0.9927); the average recovery using this method (*n* = 3) was 98.9%, the RSD (*n* = 3) was 2.9%, and RPD was 2.2%. HPLC showed that the RCE contained 3.5% salidroside (Additional file 1).

### Hypobaric Hypoxia Exposure

The method was as described previously [16]. Briefly, the male Sprague–Dawley rats (4–6 weeks old; weighing 150–180 g) were unexposed to hypoxia (control) or exposed to 50 mg/kg RCE alone. The conditions for animal exposure to hypoxia involved a simulated altitude of 8000 m for 9 h. Hypoxia-exposed rats were fed 0 (control), 50, or 100 mg/kg RCE by gavage for three days before the exposure to hypobaric hypoxia. Afterwards, rats were anesthetized and blood samples were taken from the right ventricles. Finally, the hearts were dissected and stored at −80 °C until use. All of these procedures were performed according to the Institutional Animal Care and Use Committee of the National Defense Medical Center (IACUC-15-122).

### Griess Assay

Nitrite, the breakdown product of NO, was measured using a nitrite colorimetric assay kit purchased from Cayman Chemicals (MI, USA) [11]. In brief, 20 mg sample was homogenized in 120 μl of assay buffer and then centrifuged at 10000 g for 5 min. Supernatant (100 μl) was reacted with Griess solution (100 μl) for 10 min. The absorbance was measured at 570 nm in an EIA Reader (Molecular Devices, Sunnyvale, CA, USA).

### Measurement of cGMP and Protein Carbonyl Content

Measurements of cGMP and protein carbonyl content were performed using commercial kits from Cayman Chemicals (MI, USA). Briefly, 20 mg of heart tissue were homogenized in 120 μl of 0.1 N HCl for cGMP and 40 mg of heart tissue was homogenized in 200 μl of PBS for protein carbonyl. Afterwards, the samples were centrifuged at 1000 g for 5 min. Subsequently, the supernatant was collected and measurements performed according to the manufacturer’s instructions.

### Arginase activity

Arginase activity was evaluated using a commercial kit (Abnova Corp., Taiwan) [18]. Briefly, heart tissue was homogenized and centrifuged at 10000 g for 5 min. The supernatant was reacted with reaction solution and the absorbance was measured at 570 nm.

### ROS level

The assay was performed as in our previous study [16]. Briefly, heart tissue was homogenized in RIPA lysis buffer and then centrifuged at 10000 g for 5 min. Supernatant (20 μl) was reacted with 20 mM DCFH-DA (2,7, dichlorofluorescein diacetate) in PBS at 37 °C for 15 min. The excitation wavelength was 485 nm and the emission wavelength was 538 nm.

### Western Blot Analysis

The expression of proteins in heart tissues was quantified as described previously [17]. Primary antibodies used from Cell Signaling Tech (Danvers, MA, USA) were phosphorylated eNOS (p-eNOS), Arg-1, Bcl-XL, p38 MAPK, phosphorylated p38MAPK (p-p38 MAPK), extracellular signal-regulated kinase 1/2 (ERK1/2), and phosphorylated ERK1/2 (p-ERK1/2). Primary antibodies for caspase-3, Bax, and Bcl2 were obtained from GeneTex (San Antonio, TX, USA). Antibodies for superoxide dismutase 1 (SOD1), SOD2, glutathione peroxidase 2 (GPx2), AKT, p-AKT (Thr308), p-AKT (Ser473), and eNOS were obtained from Santa Cruz (CA, USA), and antibodies for $\beta$-actin were obtained from Chemicon (Temecula, CA, USA).

### Statistical analysis

All results are presented as mean ± SEM. Significant differences between group means were determined using one-way ANOVA followed by Bonferroni’s post-hoc test. Analyses were performed using IBM SPSS Statistics version 22 (IBM® SPSS® Statistics 22). Differences were considered to be statistically significant when *p*-values were less than 0.05.

## Results

### RCE decreased the expression and activity of Arg-1 induced by hypobaric hypoxia

**Fig. 1:**
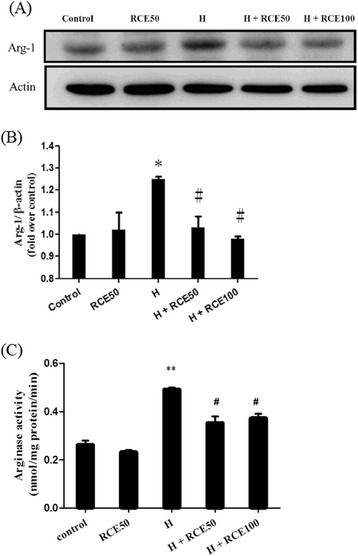
Effect of RCE on cardiac arginase expression and activity. After hypobaric hypoxia exposure (8000 m) for 9 h, the expression level of Arg-1 and arginase activity were analyzed by western blotting (**a**) and a commercial kit (**b**), respectively. A quantitative analysis of the Arg-1 expression level was conducted (**c**). The results represent the mean ± SEM (*n* = 3). *p < 0.05 and **p < 0.01 vs. the sample without RCE. #*p* < 0.05 and ##*p* < 0.01 versus hypoxia (H)

The expression of cardiac Arg-1 under conditions of hypobaric hypoxia was examined by western blotting. Hypoxia significantly (*p* < 0.05) increased the Arg-1 protein level (1.25 ± 0.01-fold over control, Fig. 1a and b). Hypoxia also significantly (*p* < 0.01) increased arginase activity from 0.26 ± 0.02 nmol/min/μl in the control group to 0.49 ± 0.01 nmol/min/μl (Fig. 1c). These results indicate that hypoxia increases both Arg-1 expression and arginase activity in heart tissue. However, treatment with RCE significantly suppressed hypoxia-induced Arg-1 expression (1.03 ± 0.05-fold over control for 50 mg/kg of RCE [*p* < 0.05] and 0.98 ± 0.01-fold over control for 100 mg/kg of RCE [*p* < 0.05]) (Fig. 1a and b). Arginase activity was significantly (*p* < 0.05) suppressed at 0.35 ± 0.03 nmol/mg protein/min for 50 mg/kg of RCE and 0.37 ± 0.02 nmol/mg protein/min for 100 mg/kg of RCE, *p* < 0.05, respectively. These findings indicated that RCE treatment diminished both hypoxia-induced Arg-1 expression and activity in rat heart.

### RCE restored NO metabolism and signaling suppressed by hypobaric hypoxia

**Fig. 2:**
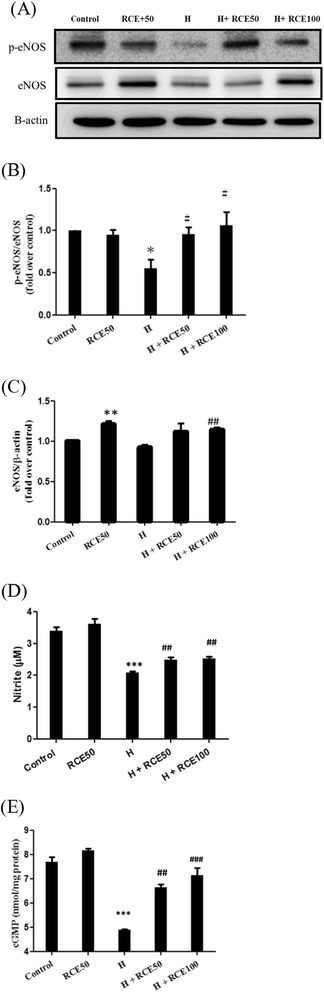
Effect of RCE on cardiac eNOS expression and downstream effectors. The expression of eNOS was analyzed by western blotting (**a**). The content of nitrite (**c**) and cGMP (**d**) was assayed by the commercial kits. A quantitative analysis of eNOS expression level was conducted (**b**). The results represent the mean ± SEM (*n* = 3). *p < 0.05 and ***p < 0.001 vs. the sample without RCE. #*p* < 0.05, ##*p* < 0.01, and ###*p* < 0.01 vs. hypoxia (H)

The effect of RCE on NO metabolism and eNOS expression was investigated. As shown in Fig. 2a and b, hypoxia significantly suppressed the phosphorylation of eNOS at Ser1177 (0.55 ± 0.11-fold of control at *p* < 0.05). However, RCE treatment restored the p-eNOS level under hypoxic conditions (0.96 ± 0.08 over the control for 50 mg/kg of RCE and 1.06 ± 0.16-fold over the control for 100 mg/kg of RCE at *p* < 0.05). RCE treatment also significantly increased the levels of eNOS under both normal conditions (1.21 ± 0.04-fold of control at *p* < 0.01) and hypoxic conditions (1.12 ± 0.10 over the control for 50 mg/kg of RCE and 1.14 ± 0.03-fold over the control for 100 mg/kg of RCE at *p* < 0.05, Fig. 2c).

In addition, RCE administration increased the nitrite content in the heart homogenate from 2.07 ± 0.04 μM up to 2.47 ± 0.07 μM for 50 mg/kg of RCE and up to 2.51 ± 0.08 μM for 100 mg/kg of RCE at *p* < 0.01, respectively (Fig. 2d). Similarly, the level of cGMP, a putative downstream product of NO, was also restored by RCE treatment from 4.89 ± 0.02 nmol/mg protein to 6.64 ± 0.13 nmol/mg protein for 50 mg/kg of RCE (*p* < 0.01) and up to 7.13 ± 0.10 nmol/mg protein for 100 mg/kg of RCE (*p* < 0.001) (Fig. 2e). Based on these findings, we inferred that RCE treatment restores the hypoxia-impaired NO bioavailability and signaling in heart tissues.

### RCE eliminated the hypobaric hypoxia-induced cardiac oxidative stress

**Fig. 3:**
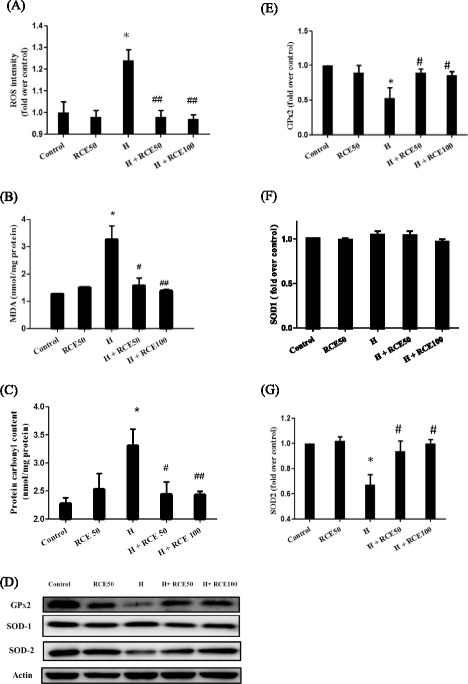
Effect of RCE on cardiac oxidative stress markers. Hypoxia-induced oxidative stress markers in the heart tissues are shown as ROS intensity (**a**), MDA content (**b**), and protein carbonyl content (**c**). The antioxidant system, SOD1, SOD2, and GPx2 were analyzed by western blotting (**d**). The quantitative analysis of SOD1 (**e**), SOD2 (**f**), and GPx2 (**g**) expression levels were conducted. Results represent mean ± SEM ($n$ = 6). *p < 0.05 vs. the sample without RCE. #*p* < 0.05 and ##*p* < 0.01 versus hypoxia (H)

In order to examine the effect of RCE on hypoxia-induced cardiac oxidative stress, the levels of ROS, MDA, and protein carbonyl in the heart tissues were measured. As shown in Fig. 3a, b, and c, hypobaric hypoxia significantly increased ROS generation (1.24 ± 0.05-fold over control at *p* < 0.05), MDA (from 1.26 ± 0.01 nmol/mg protein for the control to 3.25 ± 0.52 nmol/mg protein at *p* < 0.05), and protein carbonyl (from 2.27 ± 0.11 nmol/mg protein of the control to 3.30 ± 0.30 nmol/mg protein at *p* < 0.05) levels in hypoxic heart tissues. However, RCE treatment significantly lowered the hypoxia-induced ROS burst to 0.98 ± 0.03-fold over control for 50 mg/kg of RCE and to 0.97 ± 0.02-fold over control for 100 mg/kg of RCE at *p* < 0.01 (Fig. 3a). The lipid oxidation marker MDA was 1.56 ± 0.29 nmol/mg protein for 50 mg/kg of RCE (*p* < 0.05) and 1.37 ± 0.07 nmol/mg protein for 100 mg/kg of RCE (*p* < 0.01) (Fig. 3b). The protein carbonyl oxidation protein marker was 2.42 ± 0.23 nM/mg protein at 50 mg/kg of RCE (*p* < 0.05) and 2.43 ± 0.07 nM/mg at 100 mg/kg of RCE (*p* < 0.01) (Fig. 3c). These results indicate that RCE administration reduces hypoxia-induced cardiac oxidative stress. Interestingly, we also observed that the levels of the antioxidant enzyme SOD2 (0.67 ± 0.08-fold of the control at *p* < 0.05, Fig. 3d and g) and GPx2 (0.53 ± 0.09 fold of the control at *p* < 0.05, Fig. 3d and e) were significantly decreased in the hypoxia group compared with that in normoxia. Similarly, administration of RCE abolished the hypoxia-suppressed expressions of SOD2 (0.94 ± 0.08 and 1.00 ± 0.03-fold over control for 50 and 100 mg/kg of RCE, *p* < 0.05, respectively) and GPx2 (0.90 ± 0.05 and 0.86 ± 0.05-fold over control for 50 and 100 mg/kg of RCE, *p* < 0.05, respectively). However, no significant alteration of SOD1 expression exists under those conditions.

### RCE eliminates hypoxia-induced cardiac apoptosis

**Fig. 4:**
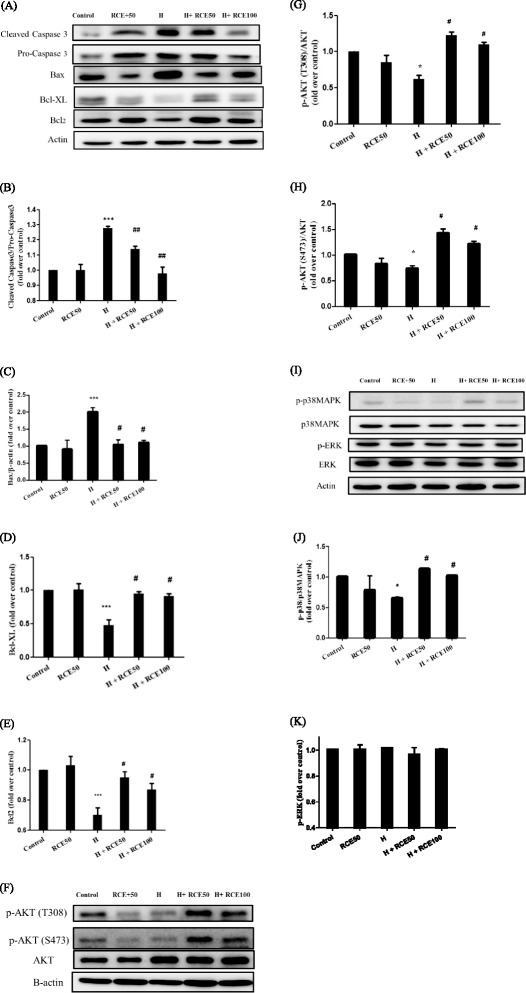
Effect of RCE on apoptotic and anti-apoptotic markers in heart tissues. The representative apoptotic and anti-apoptotic proteins were analyzed by western blotting (**a**). Quantitative analysis of caspase3 (**b**), Bax (**c**), Bcl-xL (**d**), and Bcl-2 (**e**) expression levels were conducted. Results represent mean ± SEM ($n$ = 6). *p < 0.05 vs. the sample without RCE. #*p* < 0.05 and ##*p* < 0.01 versus hypoxia (H)

In order to investigate the effect of RCE on hypoxia-induced cardiac apoptosis, the key apoptotic proteins were examined. We showed that hypoxia significantly increased the pro-apoptotic protein levels of cleaved caspase 3 (1.28 ± 0.01-fold over control, *p* < 0.05, Fig. 4a and b) and Bax (2.00 ± 0.13-fold over control, *p* < 0.05, Fig. 4a and c) and decreased the anti-apoptotic protein expressions of Bcl-xL (0.48 ± 0.08-fold over control, *p* < 0.001, Fig. 4a and d) and Bcl-2 (0.70 ± 0.05-fold over control, *p* < 0.001, Fig. 4a and e), respectively. These results indicate hypoxia causes an increase in cells undergoing apoptosis. However, RCE treatment reversed the hypoxic effects on these targets, including caspase 3 (1.14 ± 0.02 at 50 mg/kg of RCE and 0.99 ± 0.04-fold over control at 100 mg/kg of RCE at *p* < 0.05 (Fig. 4a and b), Bax (0.91 ± 0.16 and 1.06 ± 0.07-fold over control for 50 and 100 mg/kg of RCE at *p* < 0.05, respectively, Fig. 4a and c), Bcl-xL (0.95 ± 0.03 and 0.91 ± 0.04-fold over control for 50 and 100 mg/kg of RCE at *p* < 0.05, respectively, Fig. 4a and d), and Bcl-2 (0.91 ± 0.04 and 0.83 ± 0.04-fold over control for 50 and 100 mg/kg of RCE at *p* < 0.05, respectively, Fig. 4a and e).

In order to further examine the mechanism of RCE in hypoxic heart, the levels of p-AKT, p38 MAPK, and ERK were measured. Hypoxia significantly decreased the p-AKT expression at Thr308 (0.62 ± 0.05-fold over control at *p* < 0.05, Fig. 4f and g) and Ser473 (0.73 ± 0.06-fold over control, *p* < 0.05 at Fig. 4f and h) as well as p-p38 MAPK (0.65 ± 0.06-fold over control at *p* < 0.05, Fig. 4i and j). However, RCE significantly reversed the expression of p-AKT at Thr308 (1.23 ± 0.04 and 1.10 ± 0.07-fold over control for 50 and 100 mg/kg of RCE at *p* < 0.05, Fig. 4f and g) and Ser473 (1.42 ± 0.09 and 1.21 ± 0.06-fold over control for 50 and 100 mg/kg of RCE at *p* < 0.05, Fig. 4f and h) along with p-p38MAPK (1.13 ± 0.01 and 1.02 ± 0.01-fold over control for 50 and 100 mg/kg of RCE at *p* < 0.05, Fig. 4i and j). These results indicate that RCE promotes both the PI3K/AKT and p38MAPK pathways under conditions of hypoxia. However, ERK expression did not change under our experimental conditions.

## Discussion

**Fig. 5:**
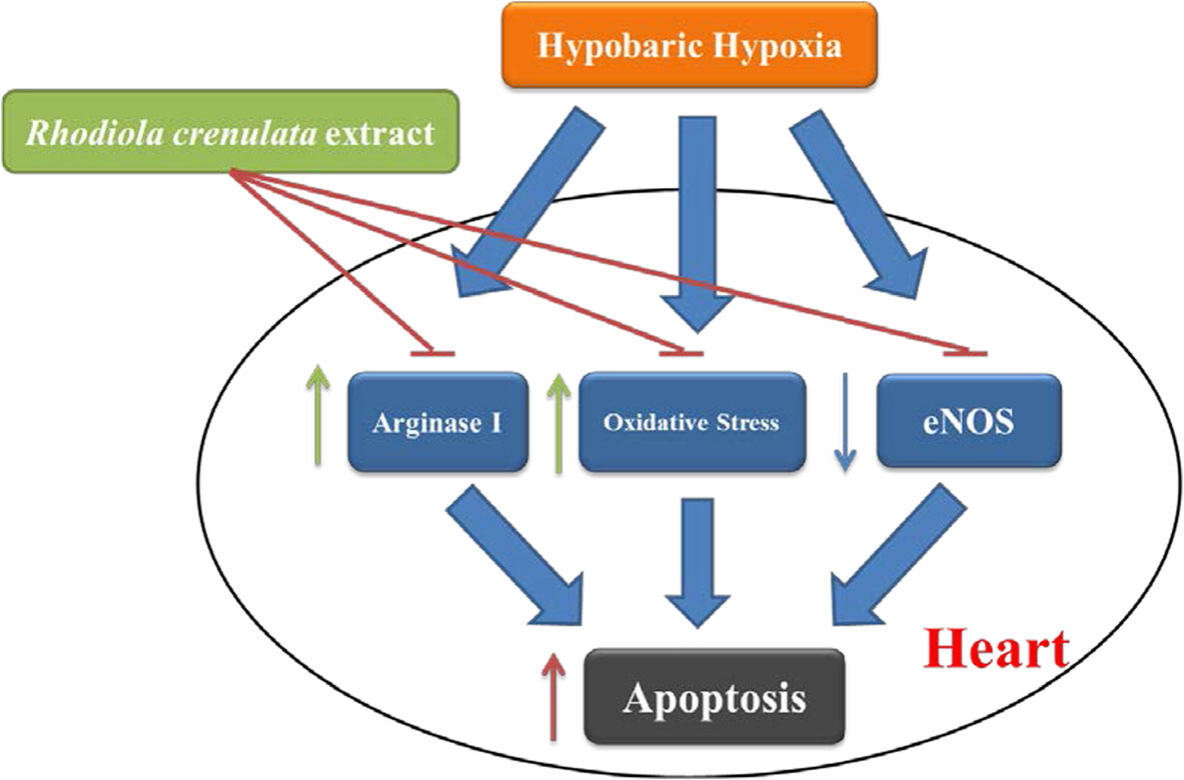
The proposed mechanism of RCE against hypoxia-induced cardiac apoptosis

The heart is very sensitive to hypoxia due to its property of high oxygen uptake [15]. Exposure to hypobaric hypoxia can consequently result in exaggerated arterial hypoxemia and lead to cardiac dysfunction including SCD [2, 19]. In this study, we showed that RCE significantly normalized the decrease in phosphorylation of eNOS produced by hypoxia. RCE also reduced Arg-1 expression level and oxidative stress markers in heart tissues under hypoxic conditions and abolished hypoxia-induced cardiac apoptosis. Taken together, these findings indicate that RCE treatment exerts cardioprotective effects in hypoxic animals by reducing hypoxia-induced oxidative stress, repressing arginase activity, and regulation of cardiac NO metabolism (Fig. 5).

The expression of eNOS and NO production in the vascular endothelium is crucial for the maintenance of vascular tone and cardiovascular physiology [20] including in the pulmonary vasculature [10]. This pathway is an oxygen-dependent process and limited by hypoxia. We showed that hypoxia decreased the expression of phosphorylated eNOS in the heart, as expected. However, this phenomenon was reversed by RCE treatment. Furthermore, the contents of cardiac nitrite and cGMP, which are markers of NO activity in biological systems [4], were restored by RCE treatment. These results indicated that RCE treatment shifted the consumption of L-arginine from Arg-1 to eNOS, increasing NO formation and its downstream effectors in the heart.

We also showed that RCE treatment affected upstream eNOS regulatory signaling by enhancing the p-AKT level under hypoxic conditions (Fig. 4f, g, and h). This is consistent with the fact that eNOS activity is regulated by protein kinase AKT in vascular endothelial cells [21] and hypoxic lung tissues [22]. Based on these findings, RCE may act to restore the expression of phosphorylated eNOS via the PI3K/AKT-associated signaling pathway in cardiomyocytes. However, this hypothesis requires further investigation. A recent study using RNA microarray technology showed that *Rhodiola* extract significantly regulates the eNOS pathway in T98G human neuroglial cells [23]. In our study, we demonstrated that RCE treatment increased both eNOS expression and NO signaling in hypoxic animals. These results might provide preliminary evidence that RCE is able to restore eNOS signaling *in vitro* and *in vivo* and that this may contribute to its ability to limit hypoxic cardiac failure.

Hypoxia has been reported to increase the expression and activity of cardiac arginase, which is highly correlated with the severity of several cardiac and vascular disorders, such as heart failure and myocardial ischemia [8]. Arginase inhibition has been shown to both improve NO availability by shifting L-arginine utilization from arginase to eNOS and to decrease oxidative stress in hypoxic heart tissue [9]. Taken together, these observations suggest that inhibition of arginase activity is a potential therapeutic strategy in hypoxia-associated heart disorders.

We showed that RCE treatment significantly decreased both the expression and activity of arginase in hypoxic heart tissue and restored NO signaling. These findings may indicate that RCE restored NO signaling partially by suppression of arginase activity, which is regulated by various upstream factors including ROS, inflammatory cytokines, and mitogen-activated protein kinase (MAPK) pathways [8].

Until now, the exact regulatory mechanism of RCE on arginase was unclear. It was reported previously that hypoxia contributed to arginase activation induced by the ROS burst through c-Jun and AP-1 interaction [9]. We have shown that RCE treatment is able to attenuate ROS intensity in both hypoxic lung [16] and heart tissues. These results suggest that RCE could inhibit arginase activity by limiting hypoxia-induced oxidative stress. Considering that arginase has been shown to be associated with many other cardiac diseases such as atherosclerosis and heart failure [8], it would be interesting to investigate the efficacy of RCE against these disorders.

Hypoxia increased cardiac oxidative stress and resulted in the impairment of NO signaling. Hypoxia also decreased the bioavailability of NO by increasing NO destruction through peroxynitrite formation as well as promoting uncoupling of eNOS [7]. These events all contribute to the pathological progression of cardiac failure [24].

Oxidative stress directly contributes to impaired cardiac-muscle contraction by modifying proteins with high levels of protein carbonyl groups [25]. In contrast, overexpression of antioxidant enzymes can improve oxidative stress-mediated cardiac defects [26]. In the present study, RCE did not change the antioxidant system under normoxia. However, in hypoxia, where SOD2 and GPx2 are decreased, RCE treatment restored expression of these fundamental cardiomyocyte anti-oxidant pathways [11, 27]. These results are also consistent with the marked radical scavenging activity of RCE reported in a previous study [28]. Together, these results suggest that RCE could decrease hypoxia-induced oxidative stress via the nonenzymatic pathway, but not by directly increasing the expression of antioxidant enzymes.

In a rodent model of exhaustive exercise, salidroside, a major bioactive compound of RCE, exerted a cardioprotective effect via its antioxidant activity [15]. In this study, we have shown that RCE reduces biomarkers of cardiac oxidative stress (ROS, MDA, and protein carbonyl) in rat myocardium. This suggests that the protective effect of RCE is associated with its antioxidant potential. In addition, growing evidence shows that obstructive sleep apnea is associated with excessive ROS production induced by hypoxia. These ROS directly contribute to decreased NO availability and eNOS uncoupling [29] as well as heart problems in the elderly [30]. It would be interesting to investigate whether RCE has beneficial effects for those populations.

Hypoxia triggers many different signaling pathways in the heart. Among them, MAPK pathways, such as ERK and p38MAPK, are of importance [31, 32]. Most studies concur that activation of p38MAPK and ERK signaling is positive for cell survival [31]. In the present study, RCE treatment significantly promoted the expression of phosphorylated p38MAPK. However, no change was observed in the level of p-ERK in our model. These results indicated that RCE promotes cardiomyocyte survival under acute hypobaric hypoxia exposure by p38MAPK, but not ERK, signaling pathway in heart tissue. These results are similar to those observed in the acute exhaustive rodent model where salidroside regulates MAPK signaling pathways, through modification of ROS generation [15]. Thus, the regulatory effect of RCE on MAPK pathways might again be due to the antioxidant property of RCE.

A recent study of note showed that oxidative stress is associated with coronary vascular tone through the AMPK-eNOS-NO pathway. This suggests a possible role of AMPK in the vascular endothelium under the hypoxic condition [30]. In our previous studies, RCE regulated both hepatic and pulmonary function via the AMPK pathway [17, 33]. It would be interesting to clarify the role of AMPK in hypoxia-treated animals in the future.

Myocyte apoptosis is a crucial modulator in the development of cardiac failure [34]. Based on this idea, a growing number of anti-apoptosis interventions for heart failure are now under investigation. The anti-cardiac apoptotic effects of RCE on chronic intermittent hypoxia in mice have been recently confirmed by Lai et al. [13, 14]. In the present study, the protective effect of RCE on acute and hypobaric hypoxia was investigated. We showed that RCE not only regulated members of the Bcl-2 family (Bcl-2, Bax, and Bcl-xL), but also the downstream markers of apoptosis, such as caspase 3 (Fig. 4). These findings indicate that RCE promotes an anti-apoptotic effect on hypoxia-treated animals, suggesting a cardioprotective effect of RCE for both acute and chronic hypobaric hypoxia exposures. It should be noted that the PI3K/AKT signaling pathway is a survival signal in response to hypoxia [35]. Thus, the increased p-AKT level conferred by RCE is consistent with the anti-apoptotic effect of RCE occurring through the activation of PI3K/AKT signaling pathway.

In addition, there are some limitations in this study. We did not monitor the blood pressure and heart rate due to the limitations of a simulated hypoxia chamber. It would be further investigated in the future study.

## Conclusion

We showed that RCE decreased cardiac arginase expression, increased NO signaling, and suppressed oxidative stress under hypobaric hypoxia conditions. This was associated with reduced hypoxia-induced cardiac apoptosis. These results might support and provide mechanistic insight into the traditional applications of *Rhodiola crenulata* for high altitude sickness.

## Additional file

Additional file 1: HPLC profiling data of salidroside (a) and RCE (b). (DOCX 120 kb)

## Abbreviations

Arg-1: Arginase 1
eNOS: Endothelial nitric oxide synthase
ET-1: Endothelin-1
GPx2: Glutathione peroxidase 2
HAPE: High-altitude pulmonary edema
MDA: Malondialdehyde
MI: Myocardial infarction
NO: Nitric oxide
PH: Pulmonary hypertension
RCE: *Rhodiola crenulata* root extract
ROS: Reactive oxygen species
RV: Right ventricular
SCD: Sudden cardiac death
SD: Sprague–Dawley
SOD1: Superoxide dismutase 1
SOD2: Superoxide dismutase 2

## References

[1] Hackett PH, Roach RC (2001). High altitude illness. N Engl J Med.

[2] Lo MY, Daniels JD, Levine BD, Burtscher M (2013). Sleeping altitude and sudden cardiac death. Am Heart J.

[3] Rimoldi SF, Sartori C, Seiler C, Delacretaz E, Mattle HP, Scherrer U, Allemann Y (2010). High-altitude exposure in patients with cardiovascular disease: risk assessment and practical recommendations. Prog Cardiovasc Dis.

[4] Calvert JW, Lefer DJ (2009). Myocardial protection by nitrite. Cardiovasc Res.

[5] Fish JE, Yan MS, Matouk CC, St Bernard R, Ho JJ, Gavryushova A, Srivastava D, Marsden PA (2010). Hypoxic repression of endothelial nitric-oxide synthase transcription is coupled with eviction of promoter histones. J Biol Chem.

[6] Wolin MS, Neo B, Patel D, Alhawaj R, Kandhi S, Ahmad M (2011). Redox regulation of responses to hypoxia and NO-cGMP signaling in pulmonary vascular pathophysiology. BMC Pharmacol.

[7] Beall CM, Laskowski D, Erzurum SC (2012). Nitric oxide in adaptation to altitude. Free Radic Biol Med.

[8] Pernow J, Jung C (2013). Arginase as a potential target in the treatment of cardiovascular disease: reversal of arginine steal?. Cardiovasc Res.

[9] Singh M, Padhy G, Vats P, Bhargava K, Sethy NK (2014). Hypobaric hypoxia induced arginase expression limits nitric oxide availability and signaling in rodent heart. Biochim Biophys Acta.

[10] Frazziano G, Champion HC, Pagano PJ (2012). NADPH oxidase-derived ROS and the regulation of pulmonary vessel tone. Am J Physiol Heart Circ Physiol.

[11] Singh M, Shukla D, Thomas P, Saxena S, Bansal A (2010). Hypoxic preconditioning facilitates acclimatization to hypobaric hypoxia in rat heart. J Pharm Pharmacol.

[12] Ma HP, Fan PC, Jing LL, Yao J, He XR, Yang Y, Chen KM, Jia ZP (2011). Anti-hypoxic activity at simulated high altitude was isolated in petroleum ether extract of saussurea involucrata. J Ethnopharmacol.

[13] Lai MC, Lin JG, Pai PY, Lai MH, Lin YM, Yeh YL, Cheng SM, Liu YF, Huang CY, Lee SD (2015). Effects of rhodiola crenulata on mice hearts under severe sleep apnea. BMC Complement Altern Med.

[14] Lai MC, Lin JG, Pai PY, Lai MH, Lin YM, Yeh YL, Cheng SM, Liu YF, Huang CY, Lee SD (2014). Protective effect of salidroside on cardiac apoptosis in mice with chronic intermittent hypoxia. Int J Cardiol.

[15] Wang Y, Xu P, Wang Y, Liu H, Zhou Y, Cao X (2013). The protection of salidroside of the heart against acute exhaustive injury and molecular mechanism in rat. Oxidative Med Cell Longev.

[16] Lee SY, Li MH, Shi LS, Chu H, Ho CW, Chang TC (2013). Rhodiola crenulata extract alleviates hypoxic pulmonary edema in rats. Evid-based complement altern med : eCAM.

[17] Lee S-Y, Lai F-Y, Shi L-S, Chou Y-C, Yen IC, Chang T-C (2015). Rhodiola crenulata extract suppresses hepatic gluconeogenesis via activation of the AMPK pathway. Phytomedicine : Int J Phycol Phycochem.

[18] Ashmore T, Fernandez BO, Branco-Price C, West JA, Cowburn AS, Heather LC, Griffin JL, Johnson RS, Feelisch M, Murray AJ. Dietary nitrate increases arginine availability and protects mitochondrial complex I and energetics in the hypoxic rat heart. The Journal of physiology. 2014;592(21):4715-31. 10.1113/jphysiol.2014.275263 PMC4253472 25172947

[19] Dehnert C, Bärtsch P, Grünig E, Mereles D (2007). High-altitude pulmonary edema and patent foramen ovale. JAMA.

[20] Naseem KM (2005). The role of nitric oxide in cardiovascular diseases. Mol Asp Med.

[21] Dimmeler S, Fleming I, Fisslthaler B, Hermann C, Busse R, Zeiher AM (1999). Activation of nitric oxide synthase in endothelial cells by aktdependent phosphorylation. Nat Genet.

[22] Kuriyama S, Morio Y, Toba M, Nagaoka T, Takahashi F, Iwakami S, Seyama K, Takahashi K (2014). Genistein attenuates hypoxic pulmonary hypertension via enhanced nitric oxide signaling and the erythropoietin system. Am J Physiol Lung Cell Mol Physiol.

[23] Panossian A, Hamm R, Wikman G, Efferth T (2014). Mechanism of action of rhodiola, salidroside, tyrosol and triandrin in isolated neuroglial cells: an interactive pathway analysis of the downstream effects using RNA microarray data. Phytomedicine : Int J Phycol Phycochem.

[24] Nojiri H, Shimizu T, Funakoshi M, Yamaguchi O, Zhou H, Kawakami S, Ohta Y, Sami M, Tachibana T, Ishikawa H (2006). Oxidative stress causes heart failure with impaired mitochondrial respiration. J Biol Chem.

[25] Tsutsui H, Kinugawa S, Matsushima S (2011). Oxidative stress and heart failure. Am J Physiol Heart Circ Physiol.

[26] Giordano FJ (2005). Oxygen, oxidative stress, hypoxia, and heart failure. J Clin Investig.

[27] Richters L, Lange N, Renner R, Treiber N, Ghanem A, Tiemann K, Scharffetter-Kochanek K, Bloch W (2011). K. B: exercise-induced adaptations of cardiac redox homeostasis and remodeling in heterozygous SOD2-knockout mice. J Appl Physiol.

[28] Chen D, Fan J, Wang P, Zhu L, Jin Y, Peng Y, Du S (2012). Isolation, identification and antioxidative capacity of water-soluble phenylpropanoid compounds from rhodiola crenulata. Food Chem.

[29] Dumitrascu R, Heitmann J, Seeger W, Weissmann N, Schulz R (2013). Obstructive sleep apnea, oxidative stress and cardiovascular disease: lessons from animal studies. Oxidative Med Cell Longev.

[30] Shafique E, Choy WC, Liu Y, Feng J, Cordeiro B, Lyra A, Arafah M, Yassin-Kassab A, Zanetti AVD, Clements RT (2013). Oxidative stress improves coronary endothelial function through activation of the pro‐survival kinase AMPK. Aging (Albany NY).

[31] Kaiser RA, Bueno OF, Lips DJ, Doevendans PA, Jones F, Kimball TF, Molkentin JD (2004). Targeted inhibition of p38 mitogen-activated protein kinase antagonizes cardiac injury and cell death following ischemia-reperfusion in vivo. J Biol Chem.

[32] Muslin AJ (2008). MAPK signalling in cardiovascular health and disease: molecular mechanisms and therapeutic targets. Clin Sci.

[33] Lee SY, Shi LS, Chu H, Li MH, Ho CW, Lai FY, Huang CY, Chang TC (2013). Rhodiola crenulata and its bioactive components, salidroside and tyrosol reverse the hypoxia-induced reduction of plasma-membrane-associated Na, K-ATPase expression via inhibition of ROS-AMPK-PKCξ pathway. Evid Based Complement Alternat Med : eCAM.

[34] van Empel VP, Bertrand AT, Hofstra L, Crijns HJ, Doevendans PA, De Windt LJ (2005). Myocyte apoptosis in heart failure. Cardiovasc Res.

[35] Dai T, Zheng H, Fu G-s (2008). Hypoxia confers protection against apoptosis via the PI3K/Akt pathway in endothelial progenitor cells. Acta Pharmacol Sin.

