# Supplementary material for: Rhodiola crenulata extract counteracts the effect of hypobaric hypoxia in rat heart via redirection of the nitric oxide and arginase 1 pathway
Source: BMC Complement Altern Med. 2017 Jan 7;17:29. doi: 10.1186/s12906-016-1524-z (PMC5219729; doi:10.1186/s12906-016-1524-z)
Supplement: Additional file 1: — HPLC profiling data of salidroside (a) and RCE (b). (DOCX 120 kb) [file 12906_2016_1524_MOESM1_ESM.docx]

**Supplementary File**

(A)


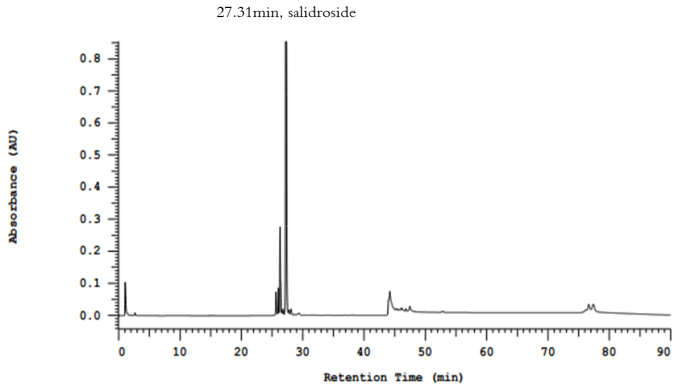


(B)


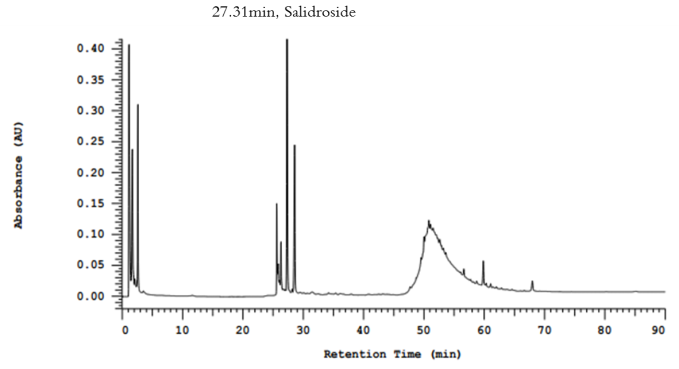


Supplementary 1. HPLC profiling data of salidroside (A) and RCE (B).

**HPLC analysis**

Constituents of *R. crenulata* ethanolic extract were analysed using a Hitachi instrument with a L-7100 series quaternary gradient pump (Hitachi, Japan), and a diode array detector (L-7455) linked to a Hitachi lachrom software data handling system (D-7000 Multi-HSM-Manager). Reverse-phase separations were carried out using a Lichro CART^®^ RP-18e column (4.0 × 250 mm i. d., 5μm; Merck, Germany). Reverse phase HPLC was performed by using the mobile phase as follows: water–acetonitrile (100:0) for 20 min, water–acetonitrile (85:15) for 15 min, water–acetonitrile (83:17) for 5 min, water–acetonitrile (50:50) for 30 min, and water–acetonitrile (0:100) for 30 min. The column temperature was maintained at 25℃. The flow rate was 1 mL/min and the injection volume was 20 μL. The eluted components were identified based on the retention time in comparison with the used reference standards. The identify of constituents was also confirmed with a photodiode array detector by comparison with UV spectra of standards over the wave length range 223 nm.
